# Current and New Biomarkers for Early Detection, Prognostic Stratification, and Management of Gallbladder Cancer Patients

**DOI:** 10.3390/cancers12123670

**Published:** 2020-12-07

**Authors:** Patricia García, Angela Lamarca, Javier Díaz, Enrique Carrera, Juan Carlos Roa, on behalf of the European-Latin American ESCALON Consortium

**Affiliations:** 1 Department of Pathology, School of Medicine, Pontificia Universidad Católica de Chile, Santiago 8330024, Chile; pgarciam@uc.cl; 2 Department of Medical Oncology, The Christie NHS Foundation Trust, Division of Cancer Sciences, University of Manchester, Manchester M20 4BX, UK; angela.lamarca@christie.nhs.uk; 3 Departamento del Aparato Digestivo, Hospital Nacional Edgardo Rebagliati Martins-Essalud, School of Medicine, Universidad Nacional Mayor de San Marcos, Lima 15081, Peru; jdiazf1@unmsm.edu.pe; 4 Department of Gastroenterology, Hospital Especialidades Eugenio Espejo, Universidad San Francisco de Quito, Quito 170136, Ecuador; ecarrera@asig.com.ec

**Keywords:** gallbladder cancer, biomarkers, genetic susceptibility, diagnosis, prognosis, patient stratification, treatment selection

## Abstract

**Simple Summary:**

Gallbladder cancer is the sixth cause of death related to digestive tract tumors, with high mortality due to delayed diagnosis at advanced stages of the disease. Moreover, treatment options for advanced gallbladder cancer usually rely on cytotoxic chemotherapy, which is frequently ineffective. Since complete surgical removal at early stages represents the best chance for curative treatment, there is an urgent need for the discovery of effective biomarkers to assess individual risk and early detection of the disease. Equally important is the development of predictive markers for adequate selection of systemic therapies to improve patient prognosis, both in the adjuvant and palliative settings. In the following review, we summarize current and newly examined biomarkers and discuss their potential utility in the management of gallbladder cancer patients.

**Abstract:**

Gallbladder cancer (GBC) is an aggressive disease that shows evident geographic variation and is characterized by a poor prognosis, mainly due to the late diagnosis and ineffective treatment. Genetic variants associated with GBC susceptibility, including polymorphisms within the toll-like receptors *TLR2* and *TLR4*, the cytochrome P450 1A1 (*CYP1A1*), and the ATP-binding cassette (ABC) transporter *ABCG8* genes, represent promising biomarkers for the stratification of patients at higher risk of GBC; thus, showing potential to prioritize cholecystectomy, particularly considering that early diagnosis is difficult due to the absence of specific signs and symptoms. Similarly, our better understanding of the gallbladder carcinogenic processes has led to identify several cellular and molecular events that may influence patient management, including HER2 aberrations, high tumor mutational burden, microsatellite instability, among others. Despite these reports on interesting and promising markers for risk assessment, diagnosis, and prognosis; there is an unmet need for reliable and validated biomarkers that can improve the management of GBC patients and support clinical decision-making. This review article examines the most potentially significant biomarkers of susceptibility, diagnosis, prognosis, and therapy selection for GBC patients, highlighting the need to find and validate existing and new molecular biomarkers to improve patient outcomes.

## 1. Introduction

Gallbladder cancer (GBC) is one of the most prevalent biliary tract cancer (BTC) and the sixth most common gastrointestinal cancer worldwide [1,2]. This aggressive disease shows evident geographic variation, ranking 20th in incidence and 17th in mortality globally, and representing 1.3% of all cancers [3]. The geographic areas with the highest mortality rates include Chile, Bolivia, Korea, Nepal, Bangladesh, Japan, Peru, Czech Republic, and Slovakia [4]. The main risk factors include cholelithiasis, gallbladder wall calcification, gallbladder polyps >10 mm, primary sclerosing cholangitis, an anomalous junction of the pancreaticobiliary duct, smoking, and obesity [2]. Therefore, this disease is strongly related to environmental and genetic factors [5], some of which are potentially modifiable.

GBC is characteristically diagnosed at advanced stages and treatment strategies remain largely ineffective, contributing to the poor prognosis of this disease [6]. Indeed, global 5-year survival for GBC and other BTCs is 5–15% (all stages jointly analyzed) [7,8]. Thus, the lack of specific symptoms at early stages and effective diagnostic biomarkers results in only 15% of patients being candidates for curative resection at the time of diagnosis [9]. On the other hand, palliative systemic therapy is the most likely treatment option for the majority of patients with locally advanced or metastatic disease [10,11,12,13].

One of the major challenges to provide adequate management options for GBC patients has been the discovery and validation of novel diagnostic and prognostic biomarkers. Currently, there exists limited and inconclusive evidence supporting the use of diagnostic biomarkers, which has resulted in relying on epidemiological associations using clinical and demographic data to stratify high-risk populations of stone-associated GBC. Therefore, our knowledge in most well-known prognostic factors has derived from the information present in complete surgical pathology reports. In terms of therapy, predictive and prognostic markers are urgently needed. In the setting of advanced disease, the access to good-quality tissue, sufficient for biomarker discovery and validation [14], together with limited translational research performed as part of prospective clinical trials, represent significant ongoing challenges [15]. In addition, the absence of actionable targets (excluding HER2) poses a major hindrance to the design of tailored pharmacological strategies beyond conventional therapy [16].

Herein, we summarize some of the most significant biomarkers for susceptibility, diagnosis, prognosis, and therapy selection of GBC patients, highlighting the need for discovery and validation of existing and novel molecular biomarkers to improve patient outcomes.

## 2. Genetic Susceptibility Biomarkers

Different factors have been associated with an increased risk of developing GBC, such as gallstone disease, female gender, and excess body weight [17]. However, the multiplicity of environmental and lifestyle factors linked to a relatively high GBC risk make it difficult to infer causality.

Great efforts have been made to establish molecular biomarkers able to identify susceptible populations. Most of the studies related to GBC susceptibility have focused on the evaluation of the inflammatory pathway, drug metabolism and hormonal pathways, as well as DNA repair and apoptosis pathways. More recently, the advent of new technologies like genome-wide association studies (GWAS), have allowed the comprehensive identification of genetic variants associated with GBC susceptibility in different populations [18].

In regards to the inflammation pathway, prostaglandin-endoperoxide synthase (PTGS) polymorphisms have been associated with increased bile duct cancer risk [19], and the frequency of the variant *PTGS2*−1195GA genotype has been associated with a significant increased GBC risk in North Indian populations (odds ratio (OR) = 2.00, 95% confidence interval (CI): 1.2–3.3, *p* = 0.006) [20]. Similarly, Toll-like receptor (TLR) variants *TLR2*—196 to 174 ins>del (*TLR* delta22) and *TLR4* Ex4+936C>T (rs4986791) have been associated with GBC susceptibility in the Indian population (OR = 1.54/1.96, 95% CI = 1.02–2.24/1.11–2.26, *p* ≤ 0.05) [21]. More recently, circulating inflammatory proteins have been associated with increased GBC risk compared to patients suffering from biliary lithiasis [22]. Koshiol et al. [22] identified a group of GBC-linked proteins, encompassing soluble TNFR2 (sTNFR2), IL-6, sTNFR1, CCL20, VCAM-1, IL-16, and G-CSF. From this list, IL-6, IL-16, CCL20, and sTNFR1 were identified as relevant in a multivariate model, which contributed to create an inflammatory score. This score was strongly associated with GBC risk compared to patients with gallstones (quartile 4 versus 1 OR for early GBC cases: 42.01, 95% CI: 4.65–379.25). However, the limited number of early GBC patients (*n* = 32), together with the lack of validation, make these results inconclusive and highlight the need of additional studies before clinical use [22].

When focusing on the genetic pathway of drug metabolism, genetic polymorphisms in the Phase I metabolizing enzyme cytochrome P450 1A1 (CYP1A1), and the Phase II drug-detoxification enzyme glutathione-S-transferase class Mu (GSTM1) are the most studied with respect to GBC risk. Among the *CYP1A1* variants, Ile462Val (rs1048943) has been associated with increased GBC risk in women of Hungarian (OR = 8.9, 95% CI: 2.9–27.4, *p* < 0.001) [23] and Japanese (OR = 2.70, 95% CI: 1.14–6.40, *p* < 0.05) [24] origins. This allele variant has been associated with increased CYP1A1 enzymatic activity, possibly leading to increased conversion of estradiol to 2-OH-E2, suggesting that the female hormone estrogen may facilitate GBC development [25]. Other studies have also evaluated this polymorphism in Bolivian [26], North Indian [27], and Chilean populations [28], where no relationship was found regarding GBC risk. Another *CYP1A1* genotypic variant, the IVS1 + 606 (rs2606345) T allele has been associated with GBC and BTCs [25]. This study included 237 GBC cases and 737 non-cholecystectomized controls from Shanghai, China. The analysis showed that carriers of the T allele (versus the GG genotype) of the *CYP1A1* IVS1 + 606 marker had a 2-fold risk of GBC (95% CI: 1.3–3.1). Interestingly, the effect of this variant on GBC risk was more pronounced among lean patients (body mass index < 23; OR = 3.3, 95% CI: 1.8–6.1, *p* interaction = 0.001) [25]. Regarding GSTM1, results have shown that relationships between genetic variants and increased risk of GBC vary among different populations. For instance, the *GSTM1* null genotype has been related to GBC risk in the Bolivian population, where the frequency was significantly higher in GBC patients than healthy controls (OR = 2.35, 95% CI: 1.03–5.37) [26]. However, independent studies reported that the frequency of *GSTM1* null genotype did not associate with higher GBC risk in Indian [27,29], Japanese [24], or Hungarian [23] populations. Future studies including a larger number of cases and controls are warranted to clarify whether these metabolism-associated genetic predispositions are common factors in GBC development across populations of divergent ancestries.

Regarding the DNA repair genetic pathway, many studies have evaluated the effects of *TP53* polymorphisms in GBC risk, considering that the *TP53* tumor suppressor gene is involved in different steps of carcinogenesis. Thus, the minor allele of *TP53* Arg72Pro polymorphism has been found to contribute to an increased risk of GBC among Japanese men (OR = 4.32, 95% CI: 1.08–17.2) [24] and an increased risk of non-adenocarcinoma GBC in the Hungarian population (OR = 3.8, 95% CI: 1.2–12.8) [23]. On the other hand, the CC genotype of the *TP53* rs1042522 polymorphism is associated with an increased risk of GBC in North Indians (age- and sex-adjusted OR = 2.81, 95% CI: 1.19–6.61, *p* = 0.02), although no association has been found in populations from Bolivia and Chile [26,28]. Other genetic variants involved in the DNA repair pathway that have been associated with an increased risk of GBC encompass Asp312Asn in the excision repair cross complementary group 2 (*ERCC2*) gene, IVS1 + 9G>C in the MutS homolog 2 (*MSH2*) gene, Ser326Cys in the 8-oxoguanine glycosylase (*OGG1*) gene [30], and the EX5-25C>T in the O6-akyl guanine DNA acyltransferase (*MGMT*) gene [31].

In terms of the apoptosis pathway, members of the major extrinsic apoptotic pathway, such as death receptors and their ligands, have been studied. Among them, an Indian study showed that the frequencies of the death receptor *DR4* haplotypes C_rs20575_A_rs20576_A_rs6557634_, G_rs20575_A_rs20576_G_rs6557634_, and G_rs20575_C_rs20576_G_rs6557634_ were significantly higher in GBC as compared to controls (OR = 2.76, 95% CI: 1.71–4.47; OR = 2.09, 95% CI: 1.21–3.62; and OR = 2.80, 95% CI: 1.16–6.76, respectively). Moreover, the stratification of subjects on the basis of gender showed that the C_rs20575_A_rs20576_A_rs6557634_ and G_rs20575_A_rs20576_G_rs6557634_ haplotypes were significantly associated with a 2-fold increased risk of GBC in females (OR = 2.60, CI: 1.49–4.52 and OR = 2.10, CI: 1.06–4.17, respectively) [32]. On the other hand, low penetrance variants in the caspase 8 *(CASP8)* gene may affect susceptibility to GBC genotypes, as demonstrated by the evaluation of genotypes and haplotypes of *CASP8* polymorphisms in the North Indian population [33].

One of the first GWAS analyses in GBC was conducted in a Japanese cohort of 41 GC patients and 866 controls, which identified 130 single nucleotide polymorphisms (SNPs) that showed a suggestive association with GBC. These SNPs were further examined in a validation cohort of 30 cases and 898 controls, where the SNP rs7504990 found in the deleted in colorectal cancer (DCC, 18q21.3) region showed a significant genome-wide association with GBC susceptibility in the Japanese population (OR = 6.95, 95% CI: 3.43–14.08, combined *p* = 7.46 × 10^−8^) [34]. Another case-control GWAS conducted in a discovery cohort of 1042 GBC cases and 1709 controls from predominantly North and Northeastern Indian populations, identified a strong association between common genetic variants in the chromosomal region 7q21.12, responsible for both the *ABCB1* and *ABCB4* genes, and risk of GBC. The most notable SNPs after replication and meta-analysis were rs1558375 (GWAS *p* = 3.8 × 10^−9^; replication *p* = 0.01; combined *p* = 2.3 × 10^−10^), rs17209837 (GWAS *p* = 2.0 × 10^−8^; replication *p* = 0.02; combined *p* = 2.3 × 10^−9^), and rs4148808 (GWAS *p* = 2.4 × 10^−8^; replication *p* = 0.008; combined *p* = 2.7 × 10^−9^) [35]. Interestingly, these *ABCB1/4* GBC risk variants also showed similar risk effects in Chileans, according to a recent multicenter population-based candidate variant association study that included 255 GBC cases and 2042 controls from a Chilean retrospective study [36].

Genetics variants within the *ABCG8* and *TRAF3* genes have been reported to confer GBC risk development in Chilean population [37]. In this study, the discovery GWAS stage involved 529 gallstone disease (GSD) cases and 566 controls from admixed Chilean Latinos with Mapuche Native American ancestry. After validation of the top-ten candidate variants in an independent cohort, composed of 1643 individuals (626 GSD cases and 1017 controls), only selected variants within the *ABCG8* (rs11887534, OR = 1.59, 95% CI: 1.20-2.11, *p* = 0.001) and *TRAF3* (rs12882491, OR = 1.30, 95% CI: 1.09–1.54, *p* = 0.003,) genes were associated with GSD. A subsequent examination of these variants in a cohort of 397 GBC patients revealed that both SNPs were associated with this pathology (*ABCG8* rs11887534: OR = 1.77, 95% CI: 1.27–2.45, *p* = 6.9 × 10^−4^; *TRAF3* rs12882491: OR = 1.24, 95% CI: 1.004–1.53, *p* = 0.045) [37].

Table 1 summarizes the major genetic susceptibility biomarkers described for GBC in different populations.

## 3. Diagnosis Biomarkers

To date, there exist no specific tumor markers for early diagnosis of gallbladder cancer [38]. As early GBCs do not exhibit specific signs and symptoms, they are most often discovered incidentally after cholecystectomy. Therefore, tumor markers are usually not available from the preoperative assessment [39]. Symptoms usually are indistinguishable from those produced by gallstones. Palpable mass, hepatomegaly, and jaundice are frequently found in advanced stages. Ultrasound may be the initial examination for a patient with gallbladder carcinoma who presents with abdominal distension or right upper quadrant pain [40]. Other imaging techniques, such as ultrasonography (US), computed tomography (CT), and magnetic resonance imaging (MRI), are also usually used to diagnose GBC when patients develop signs or symptoms suggestive of cancer [41]. Real-time elastography (RTE) is turning into a promising parameter, that differentiates malignant tissue from benign tissue relative to its rigidity [42] with a striking specificity and sensitivity of 100%. RTE with contrast is important in early imaging diagnosis; it has been suggested that its combined use with circulating biomarkers may aid in the timely diagnosis of this malignancy [43].

In terms of early diagnosis biomarkers, circulating markers (easily accessible through, for example, a non-invasive blood sampling) are the ones with most interest to be developed. In this context, liquid biopsy has been proposed as a promising noninvasive strategy to support early diagnosis during surveillance of higher risk patients [5,44]. However, very few data are currently available, and its clinical applicability has not been established. A study by Kumari et al. investigated the level of circulating serum free DNA (cfDNA) in GBC to determine its role in diagnosis. They compared the cfDNA levels between 34 cases of GBC, 22 cases of cholecystitis, and 17 healthy controls using quantitative PCR (qPCR). The results showed that cfDNA was significantly higher in cancer patients as compared to the cholecystitis and control group [45]. Moreover, a cut-off value of cfDNA at >218.55 ng/mL discriminated cancer patients from healthy controls with 100% sensitivity (95% CI: 89.6–100; *p* < 0.001) and 100% specificity (95% CI: 80.3–100; *p* < 0.001), whereas a cfDNA value at >372.92 ng/mL differentiated cases of cholecystitis and cancer patients with 88.24% sensitivity (95% CI: 72.5–96.6; *p* < 0.001) and 100.00% specificity (95% CI: 84.4–100.0; *p* < 0.001) [45]. Recently, a subsequent study reported the diagnostic utility of detecting long DNA fragments (ALU247) in serum samples. At a cut-off point of >406.5825 ng/mL, ALU247 discriminated GBC (*n* = 60) from controls (*n* = 36) with a sensitivity, specificity, and diagnostic accuracy of 80.0%, 86.1%, and 82.2%, respectively [46]. The detection of circulating tumor DNA (ctDNA) in bile has been also suggested as a possible diagnostic marker. Kinugasa et al. used next generation sequencing (NGS) to analyze mutations in DNA isolated from bile and tumor tissue of 30 patients with GBC. The mutation concordance rate between GBC tissue DNA and bile ctDNA was 85.7%, although the mutation frequencies in ctDNA were approximately half of those detected in tumor tissue DNA [47]. Similarly, Shen et al. reported a correspondence between molecular features detected in bile and tissue sampling obtained from BTC patients. They used targeted deep sequencing and compared bile cfDNA and tumor DNA for single nucleotide variation (SNV)/insertion and deletion (Indel) and copy number variation (CNV), revealing a high sensitivity (94.7% and 75%, respectively) and specificity (99.9% and 98.9%, respectively) [48]. Although these studies propose cfDNA and ctDNA as promising liquid biopsy-based tools for GBC diagnosis, prospective studies on larger cohorts of patients at different stages of GBC are needed to confirm the above results and establish the use of those markers in clinical practice.

Regarding already existing tumor biomarkers, cancer antigens are the ones that have been most studied. Particularly carbohydrate antigen CA 19-9, which has shown significant diagnostic and prognostic value for cholangiocarcinoma [49] and pancreatic cancer [50]. However, the diagnostic value of CA 19-9 for GBC has not been validated. Wang et al. reported that serum CA 19-9 levels are higher in patients with GBC than in those with benign gallbladder disease and healthy controls, showing a sensitivity of 71.7% and a specificity of 96.1% when used as an individual marker [51]. The same study reported the diagnostic value of CA 242, a cancer antigen that is not affected by inflammatory disorders. This marker showed remarkable specificity of 98.7% for the diagnosis of GBC. The combination of CA 19-9, CA 125, and CA 242 increased the diagnostic specificity, but not its sensitivity, reaching 100% specificity with a positive predictive value (PPV) of 100% [51]. Previously, Rana et al. reported that CA 242 has a high specificity and PPV for the diagnosis of GBC, and high discriminatory potential for differentiating malignant from benign biliary disease [52]. The performance of CA 242 as a diagnostic test was superior when compared to carcinoembryonic antigen (CEA) and CA 19-9, which showed a sensitivity, specificity, PPV, and negative predictive value (NPV) of 64%, 83%, 88%, and 53%, respectively [52]. However, the diagnostic accuracy of these markers needs to be evaluated in larger subset of patients from different populations.

Although the diagnostic value of CA 19-9 in GBC is controversial, it has recently been proposed as a predictor of resectability in patients with radiologically resectable GBC. Liu et al. [9] analyzed the preoperative serum levels of CA 19-9 in 292 patients with surgical treatment and determined a cut-off point of 98.91 U/mL in the prediction of resectability (R0 resection), with a sensitivity of 76.3% and specificity 70.8%. Higher values were considered indicative of unresectability [9].

Another promising candidate is the serum-soluble fragment of cytokeratin 19 (CK 19), CYFRA 21-1, that is associated with tumor progression and poor postoperative outcomes in patients with intrahepatic cholangiocarcinoma [53]. Serum tests in GBC patients have shown that CYFRA 21-1 is a promising biomarker for GBC, with a sensitivity of 93.7% and specificity of 96.2% [54]. According to this study, CYFRA 21-1 also correlated with tumor aggressiveness, prognosis, and recurrence-free survival.

Table 2 summarizes the major diagnosis biomarkers described for GBC.

Since, as mentioned above, GBC is, on many occasions, an incidental finding following cholecystectomy, researchers have also focused on the identification of biomarkers within the resection specimen that could maybe associate with the histogenic malignant transformation. Multiple studies have been conducted to assess early detection strategies during the characteristic dysplasia-carcinoma sequence. Progression from dysplasia has shown a strong relationship with *KRAS* and *TP53* mutations in tumor tissues [5,55]. These mutations are considered as potential early diagnostic and therapeutic targets [56]. GBC displays a basic inflammatory pattern, resulting in the elevation of proteins called neurotrophins, which have been analyzed by immunohistochemistry. Such analyses showed an increase in the nerve growth factor (NGF) and the neurotrophic tyrosine kinase receptor TrKA in gallbladder cancer glands. Additionally, these have been associated with the expression of tumor markers such as MB1, CD34, and CA 153 with a significant role in prognosis, progression, and discrimination between benign and malignant lesions [57]. It has also been determined that the elevation of the neutrophilic lymphocyte radius (NLR) in combination with CA 19-9 shows a better diagnostic efficiency to differentiate benign from malignant lesions, for which they could be used as serum biomarkers for early cancer [58].

## 4. Prognostic Biomarkers in GBC

Prognosis of GBC is largely influenced by the disease stage at the time of diagnosis. Therefore, the most described prognostic factors are those related with the clinical stages and some pathological parameters. Many studies have established their clinical relevance, focused on the elaboration of scoring systems that allow the accurate stratification GBC patients into prognostic categories. A plethora of molecular markers have been identified and proposed as novel candidates with prognostic value, yet they have not been incorporated into clinical practice. Some of the main pathological and molecular prognostic markers reported for GBC are reviewed in this section.

### 4.1. Pathological Prognostic Markers for Early and Advanced Gallbladder Cancer

The most used staging system worldwide is the tumor-node metastasis (TNM) of the International Union Against Cancer (UICC) and American Joint Committee on Cancer (AJCC), which plays a critical role in predicting patient prognoses. Accordingly, a higher T category and the presence of lymph node metastasis have been reported as strong predictors of poor survival [59,60,61]. Carcinomas confined to—and above—the tunica muscularis (Tis, T1a, or T1b), often grouped as “early gallbladder carcinomas”, show high survival rates of about 80% or higher [62,63,64,65,66,67]. For these cases, simple cholecystectomy is considered curative [39]. However, the management of T1b disease is somewhat controversial due to the variability observed in survival rates, which range from 37.5% to 100% [68]. Thus, whereas the National Comprehensive Cancer Network (NCCN) guidelines recommend radical resection along with portal lymph node dissection for T1b GBC [69], the Korean and Japanese guidelines recommend simple cholecystectomy [70,71]. Recently, an international multicenter study analyzed the clinical outcomes of 237 patients with T1b GBC according to the type of surgery (simple versus extended cholecystectomy), and showed that there exist no significant differences in the 5-year overall disease specific survival (DSS) between both treatments, concluding that extended cholecystectomy is not needed for the treatment of T1b GBC [72]. Consistent with this report, Yuza et al. [69] evaluated the long-term survival benefit of a surgical procedure in 47 Japanese patients with T1b GBC, showing that both OS and DSS between simple cholecystectomy and radical resection are comparable [69]. A factor that most likely contributes to the conflicting impressions regarding the optimal management of early GBC is the understaging phenomenon that occurs when the surgical specimen is not fully examined (mapped). The random sampling protocols employed by most Western countries may explain the low survival rates reported in some studies [68,73]. In contrast, studies using surgical specimens subjected to precise pathological examination and mapping of cancer lesions have reported survival rates over 90%, even for T1b cases [69,72,74].

For early GBC, poor and undifferentiated tumors are associated with worse survival [67,75]. Another histological aspect representing an adverse prognostic value and that may serve to identify patients more prone to disease recurrence, is the presence of deep invaginations of the lamina propria and the epithelium itself, known as Rokitansky–Aschoff sinuses [63,74,76]. Roa et al. [74] reported that T1a and T1b patients with involvement of Rokitansky–Aschoff sinuses had a significantly shorter survival and were much more likely to die of GBC, with an odds ratio of 7.3 [74]. However, despite being an independent predictor of clinical outcome, this feature is not included in the current TNM staging of early GBC.

Prognosis is less favorable for patients with cancers invading beyond the muscular layer, particularly for patients at stages IIIB or higher [62]. In this regard, residual disease is widely considered a negative prognostic factor for incidental GBC. Therefore, re-exploration and definitive resection are recommended for selected patients with invasive tumors without evidence of disseminated disease. Single and multicenter studies have shown that residual disease is found in over 50% of incidental GBC, involving mainly lymph nodes, common bile duct, and the liver [65,77,78,79,80]. The presence of residual disease is significantly associated with a higher T, TNM stage [78,79,81], and disease recurrence [78]. Moreover, residual disease at local or regional sites predicts DSS, independently of all other tumor prognostic variables [78,81]. Recently, Gil et al. [81] analyzed patterns of residual disease in patients undergoing re-resection for incidental GBC in two South American referral centers. This study reported a significant reduction in DSS comparing patients with and without residual disease (19.6 months vs. 62.7 months, *p* < 0.001, respectively) [81].

Considering the impact of residual disease in the outcome of GBC patients, the complete removal of any clinically evident tumor lesion and the ability to achieve negative surgical margins represent the only curative option. For incidental GBC, a re-resection to eradicate areas infiltrated by locoregional residual disease may significatively improve the survival rates of T2 and T3 patients [65]. In fact, studies have reported that the 5-year survival is less than 20% for T2 patients who underwent simple cholecystectomy, but can reach 60% to 90% when radical resection is performed [82]. However, the need of extended surgery for T2 tumors should consider the tumor location, as studies have reported that hepatic resection is not a determinant of better survival for peritoneal-side T2 GBC (pT2b) [83]. In a recent study, Kim et al. [83] evaluated the clinicopathological factors affecting survival outcomes of patients at stage T2a (*n* = 82) and T2b (*n* = 30) who underwent curative resection. This analysis showed no differences in survival rates between the two groups, according to whether regional lymphadenectomy or hepatic resection was performed. However, in the T2b group, patients who underwent hepatic resection had better survival rates than those who did not [83].

Given the differences among survival rates beyond the T-stage, the identification of patients at high risk of recurrence and the implementation of postoperative surveillance strategies are essential for improving patient outcome, especially for incidental GBC. Some studies have identified tumor grade, lymphovascular invasion, and perineural invasion as independent prognostic factors that also correlate with the presence of residual and/or disseminated disease at the time of re-resection [83,84,85,86,87,88]. Recently, the U.S. Extrahepatic Biliary Malignancy Consortium (USEBMC) developed a Gallbladder Cancer Predictive Risk Score (GBRS) based on four pathology-derived risk factors: T-stage, tumor grade, lymphovascular invasion, and perineural invasion. Patients were categorized into low, intermediate, and high-risk groups based on their total risk score. The analysis of 88 patients showed that each progressive GBRS group was significatively associated with i) an increased prevalence of locoregional residual disease and distant disease at the time of reoperation, and ii) a significant decrease in overall survival (OS) [86]. Moreover, the GBRS was a stronger predictor of locoregional residual disease and distant disease, as the odd ratio comparing high to intermediate GBRS groups was 4.5 (95% CI: 1.7–11.6; *p* = 0.002) and 12.2 (95% CI: 1.5–100.0; *p* = 0.02), respectively [86]. Mochizuki et al. [87] evaluated the prognostic ability of the GBRS in a single-center cohort of 56 GBC patients following curative surgery and determined that higher GBRS associated with poor long-term prognosis and high rate of tumor recurrence in incidental and non-incidental GBC [87]. Similarly, the GBRS was useful to predict the presence of regional or distant residual disease in a subgroup of 25 patients with incidental GBC, which was significantly higher in high-risk patients than in intermediate-risk patients (80% vs 30%, *p* = 0.041) [80]. Those studies underline the potential value of this novel predictive risk-score to guide treatment strategies regarding the selection of patients for reoperation and adjuvant therapy.

### 4.2. Molecular Prognostic Biomarkers

In addition to the well-known pathological prognostic factors, many molecular features associated with GBC pathogenesis have been proposed as potential biomarkers that might help stratify patients with GBC. Thus, overexpression of p53 [89,90,91], mutations in *KRAS* [92], amplification of HER2/neu [91,93], overexpression of EGFR [94,95], among other potential molecular prognostic factors such as microRNAs (recently reviewed by Montalvo-Jave et al. [5]) have been associated with worse prognosis. Most potential markers have been evaluated in formalin-fixed paraffin-embedded (FFPE) cancer biopsy sections by immunohistochemical analysis, but there are also promising candidates based on RNA/DNA and serum protein detection. A selection of potential independent molecular prognostic markers is listed in Table 3.

Despite the potential of these biomarkers, they have yet to be validated in independent cohorts. Therefore, they have not yet passed the discovery phase and are not ready for use in clinical practice. Clinical implementation depends mainly on the added clinical value that the validation phases provide, but the lack of samples with adequate clinical follow-up, robust screening tests, and financial resources make it difficult to move forward in solving this problem.

Given the low global incidence of GBC, most studies group GBC with other biliary tract cancers (BTCs), such as cholangiocarcinoma. In this context, the technological progress in next-generation sequencing (NGS) has facilitated the characterization of the genomic landscape of BTCs and provided new options for the discovery of biomarkers for clinical oncology. Those studies have shown that the most frequently altered genes in GBC are *TP53*, *CDKN2A/B*, *ARID1A*, *ERBB2*, and *PI3KCA* [113,114,115,116,117]. Some series have also reported *KRAS* [113,115] and *SMAD4* [115,118] as commonly mutated genes in this neoplasia. Recently, Wardell et al. investigated somatic and germline mutations in 412 BTCs from Japanese and Italian populations, which included 66 gallbladder or cystic duct cancers. They performed whole-exome sequencing (WES), whole-genome sequencing (WGS), as well as targeted sequencing, and identified 32 significantly and commonly mutated genes in BTC including *TP53*, *KRAS*, *SMAD4*, *NF1*, *ARID1A*, *PBRM1*, and *ATR*. Gallbladder subtypes were significantly enriched for *TP53* mutations. Both univariate and multivariate analysis showed strong negative effects on OS in BTC patients harboring mutations in *ARID1A* (*n* = 22, *p* = 0.0011) and *KRAS* (*n* = 63, *p* = 0.0042) [119]. Bagante et al. [120] also reported the clinical value of genetic classification systems by studying the association between two gene mutation panels and the prognosis of BTCs patients undergoing curative intent resection. A total of 71 patients were stratified into two groups based on the mutational burdens: *IDH1-2*/*BAP1*/*PBRM1* (*n* = 23; 32.4%), and *KRAS*/*TP53* (*n* = 48; 67.6%). The last group was enriched for individuals with a diagnosis of perihilar cholangiocarcinoma (PHCC) and GBC (*n* = 38; 79.2%), whereas patients with intrahepatic cholangiocarcinoma (ICC) were more likely to harbor *IDH1-2*/*BAP1*/*PBRM1* mutations (*n* = 14; 60.9%). This genetic classification was strongly associated with survival, showing that patients in the *IDH1-2*/*BAP1*/*PBRM1* group had a 5-year OS of 39.5% (95% CI: 18.4–60.0), versus 13.0% (95% CI: 4.3–26.8) for patients in the *KRAS*/*TP53* group (*p* = 0.032). Moreover, patients in the *KRAS*/*TP53* group had a 2-fold increased risk of death compared with patients in the *PBRM1/IDH1/BAP1* group (*p* = 0.028), confirming the strong prognostic role of genetic mutation profiling and classification approaches [120].

## 5. Prognostic and Predictive Biomarkers for Treatment Selection

Treatment options for advanced gallbladder cancer usually rely of cytotoxic chemotherapy [121,122,123], since the targeted therapies such as fibroblast growth factor receptor (FGFR) and isocitrate dehydrogenase (IDH)-1 inhibitors are of limited use in this setting [16]. Recent data suggest that advanced GBC may have a worse prognosis compared to other BTCs when treated with standard of care chemotherapy [124,125], highlighting the importance of adequate treatment selection for this patient population.

### 5.1. Predictors of Response to Adjuvant Treatment

Currently, standard of care adjuvant treatment (capecitabine) is based on results from the BILCAP trial [126]. Few publications are available exploring potential predictive factors of response to capecitabine in GBC. The expression of thymidine phosphorylase is considered one of the potential predictive biomarkers to response to capecitabine [127]. A higher expression of thymidine phosphorylase has been shown in GBC (vs. other BTCs), which seemed to be linked to a worse prognosis and more advanced stages [128]. However, its role as a predictive factor of benefit from adjuvant capecitabine is still to be confirmed and further studies are required.

Retrospective studies have also explored the potential predictive impact of excision repair cross-complementation group 1 (ERCC1) and X-ray repair cross-complementing group 1 (XRCC1) in tumor samples from patients with GBC (and other BTCs) undergoing gemcitabine as adjuvant therapy [129]. This study showed that high nuclear expression of both proteins was able to predict better overall survival [129].

### 5.2. Predictors of Response to Palliative Chemotherapy

The most active chemotherapy compounds for advanced GBC include gemcitabine and platinum agents [130,131]. Current standard of care treatment is cisplatin and gemcitabine (CisGem), based on results from the ABC-02 clinical trial [121]. Following progression to first line chemotherapy, evidence exists for rescue chemotherapy with 5-fluorouracil and oxaliplatin (FOLFOX) [122]. New systemic strategies include treatment with triple chemotherapy such as triple chemotherapy combinations by adding S-1 or nab-paclitaxel to the standard of care CisGem [132,133] or FOLFIRINOX (5-fluorouracil, irinotecan, and oxaliplatin) [134], within others.

Multiple studies have explored potential clinical factors associated with outcome to first-line CisGem [135,136,137,138]. Within the identified factors, poor performance status, elevated serum lactate dehydrogenase, elevated neutrophil-to-lymphocyte ratio, low hemoglobin, metastatic disease, increased bilirubin, increased neutrophils, and high baseline tumor markers were associated with poor outcome [136,137,138]. In addition, reduction in CA 19-9 during therapy predicted longer patient survival [135].

Impact of molecular biomarkers have not been so widely explored. Small series suggested that the presence of SNPs in the cytidine deaminase (CDA) gene were associated with increased efficacy of gemcitabine-based chemotherapy in BTC [139]. In addition, translational research associated to the ABC-03 clinical trial explored the impact of changes in 15 circulating plasma angiogenesis or inflammatory-related proteins and cytokeratin-18 (CK18) on CisGem-treated patients’ outcome [140]. In this study, patients with increasing levels of VEGF-A at any time had a worse progression-free survival (PFS) and OS. In addition, rising levels of CK18 and VEGFR2 during treatment were also associated with worse outcomes [140].

In view of wide use of platinum agents for management of GBC, DNA damage repair (DDR) has attracted lots of attention in BTCs as potential predictive factors to response to platinum-based chemotherapy [141,142]. Whole-exome sequencing analyses identified somatic mutations in DDR genes in BTC [143,144,145,146]. Mutations in DDR genes were explored in a series of 422 BTC (including 92 GBC) [147]; higher mutation rate in DDR genes (63%) was identified in GBC (compare to other BTCs). Other series including 623 GBC samples, have shown different results (*BRCA2* or *ATM* mutations in 7.8% of patients; TMB was low) [148]. The presence of such mutations was suggested to have an impact on response to platinum-based chemotherapy [31,149,150,151], even though such findings have still not been validated in clinical trials.

### 5.3. Response to Targeted Therapies

For many years, the use of targeted therapies was disappointing and of little benefit to patients diagnosed with BTCs. This was mainly because the studies did not follow a “precision medicine” approach and were therapies administered to “all comers” without adequate patient selection due to limited understanding of molecular biology and lack of predictive biomarkers [152]. This approach has radically changed with the development of FGFR and IDH-1 inhibitors [16].

Unfortunately, GBCs have shown absence of *FGFR* fusions and *IDH* mutations [153], in view of which, current clinical development of targeted therapies remains limited. Main targeted therapy strategies in GBC are focused on targeting HER gene family [16,114,143,144,145,152,154,155,156,157,158,159,160] and other unusual (present in <5–10% of GBC) aberrations such *BRAF* and *RNF43* mutations [161,162] or *TRK*-fusions [163].

Mutations in the ERBB family of proteins and related pathways were found in ~35% of GBC tumors [164]. In view of this, treatment with novel tyrosine kinase inhibitors and monoclonal antibody strategies targeting the HER pathway [165,166,167,168] have been developed in BTC, with special interest in GBC. 

Despite initial excitement, several clinical trials exploring HER pathway with tyrosine kinase inhibitors such as lapatinib [169,170] or erlotinib [171] in HER-overexpressing BTC and GBC patients brought disappointing results. It has been suggested that variability regarding the HER2 overexpression rate in different GBC series [160,172,173,174], together with difficulties to assess HER2 immunohistochemistry in BTC and GBC could have led to inadequate patient selection and derived on treatment failure [175]. Because of this, such molecules were tested not only in patients showing HER2 overexpression, but also HER2 mutations. Initial findings with pertuzumab combined with trastuzumab in 8 and 3 patients with HER2 amplification and HER2 mutations, respectively, reported an increased objective response rate in those patients with HER-2 mutations (7.5% vs. 33.3%) [176]. Unfortunately, when other HER inhibitors (i.e., neratinib) were tested in HER-2 mutated patients, objective response rate remained low (10%) [177]. In depth understating of the biological role of HER2, together with larger studies exploring the anti-tumor effect of HER inhibition in GBC, are required to define the role of HER2 in GBC not only as a target but also as a potential predictive factor to HER inhibitors.

Some studies are pointing to HER3 as a potential new target to develop [165,178]. Unfortunately, studies have focused mainly on HER2 expression [175], with HER3 expression being underexplored [160]. Currently, the prognostic role of HER3 in BTC remains unclear, with conflicting findings between studies [179,180].

### 5.4. Immunotherapy and Potential Predictive Factors

Immunotherapy for advanced GBC is still under development [181,182]. Therapeutic strategies such as the use of check-point inhibitors are being investigated in BTC [159]. However, GBC has rarely been represented in these studies [183] and further data would be required to assess its role. 

Even though mutational load seems to be high [159], mismatch repair and microsatellite instability are infrequent (5–13%) [184,185] in most of the series, with few exceptions [149]. Up to now, the only role of immunotherapy in GBC seems to be limited to patients with mismatch repair and microsatellite instability [183,186,187] which even though present in <5% of all patients [185,188], represents a robust predictive biomarker in solid tumors (including GBC) [189].

The main predictive biomarkers for treatment selection are summarized in Table 4.

## 6. Conclusions

**Figure 1:**
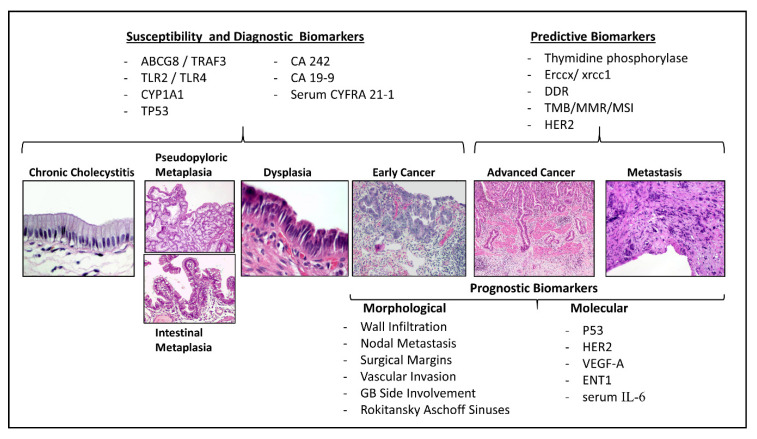
Biomarkers throughout the natural history of gallbladder cancer. Schematic representation of the most significant biomarkers according to the histogenic sequence of gallbladder cancer.

The establishment of stratification approaches capable of correctly categorizing patients according to GBC risk is an urgent necessity in order to improve early diagnosis and to support surgical decision-making. Until now, only a few examples seemed to contribute to effectively predict susceptibility or show reliability for the diagnosis of GBC (Figure 1). Despite all the advances in understanding the genetic and molecular abnormalities involved during the carcinogenesis of many solid tumors, the main elements that help prognostic stratification and management of GBC patients rely on a complete surgical report of the tumor morphological features using standardized nomenclature.

In addition, development of predictive markers for adequate selection of systemic therapies for patients with GBC, both in the adjuvant and palliative settings, is urgently required. For many years, clinical trials have lacked associated translational research endpoints and sample collection, which has led to limited understanding of factors associated with treatment success and treatment failure. Current clinical trials have been modernized and translational research is being included in many of them, incorporating both, retrospective and prospective, sample recollection. These will allow the identification of the so much needed predictive biomarkers, not only for further development of new targeted agents and immunotherapy, but also to better understand primary and acquired resistance to standard of care chemotherapy agents.

## Figures and Tables

**Table 1:** Biomarkers of genetic susceptibility in gallbladder cancer.

Genetic Variant	Measurement Technique	Utility	Reference
TLR2 (Delta22) and TLR4 (rs4986791)	PCR-RFLP	Higher susceptibility in North Indian population (233 GBC and 257 controls OR = 1.54/1.96, 95% CI: 1.02–2.24/1.11–2.26).	[21]
CYP1A1 (rs1048943)	PCR-RFLP	Increased risk of GBC among Hungarian and Japanese women (37 GBC and 48 controls; OR = 8.9, 95% CI: 2.9–27.4; and 33 GBC and 91 controls; OR = 2.70, 95% CI: 1.14–6.40, respectively).	[23,24]
CYP1A1 (rs2606345)	TaqMan assay	Higher risk and susceptibility in Chinese population (237 GBC and 737 controls; OR = 2.0, 95% CI: 1.3–3.0)	[25]
GSTM1 null genotype	PCR-RFLP	Increased risk in Bolivian population (32 GBC and 86 controls; OR = 2.35, 95% CI: 1.03–5.37)	[26]
TP53 Arg72Pro	PCR-RFLP	Increased risk of GBC among Japanese men (21 GBC and 87 controls; OR = 4.32, 95% CI: 1.08–17.2)	[24]
*MSH2* IVS1 + 9G>C, *ERCC2* Asp312Asn, and *OGG1* Ser326Cys	PCR-RFLP	Increased risk in Indian population (230 GBC and 230 controls; OR = 1.8, 95% CI: 1.1–3.1; OR = 2.1, 95% CI: 1.1–4.0; and OR = 2.5, 95% CI: 1.1 –5.4, respectively).	[30]
ABCG8 (rs11887534) and TRAF3 (rs12882491)	GWAS	Higher risk in Chilean Latinos with Mapuche Native American ancestry (397 GBC and 667 controls; OR = 1.77, 95% CI: 1.27–2.45; and OR = 1.24, 95% CI: 1.004–1.53, respectively).	[37]

Abbreviations: PCR-RFLP, polymerase chain reaction-restriction fragment length polymorphism; GWAS, genome-wide association study.

**Table 2:** Biomarkers for diagnosis.

Biomarker	Measurement Technique	Utility	Reference
CA 242	ELISA	Diagnosis (sensitivity 64%, specificity 83%; positive predictive value 88%, negative predictive values 53%)	[52]
CA 19-9	ECLIA	Diagnosis (sensitivity 71.7%, specificity 96.1%)	[51]
CYFRA 21-1	ECLIA	Diagnosis (cut-off values 3.27 ng/mL; sensitivity 93.7%, specificity 96.2%)	[54]

Abbreviations: ELISA, enzyme-linked immunosorbent assay; ECLIA, electrochemiluminescence immunoassay.

**Table 3:** Candidate molecular biomarkers for predicting prognosis in gallbladder cancer.

Biomarker	Measurement Technique	Prognostic Significance	Reference
p53	IHC	Overexpression associated with reduced survival rates (*n* = 138, 96, and 60 GBC in each study).	[89,90,91]
HER2	IHC	Overexpression associated with poor survival (*n* = 60)	[91]
EGFR	IHC	High expression was related to worse prognosis (*n* = 34 and 39 in each study)	[94,95]
VEGF-A	IHC	High expression associated with a poor prognosis in advanced GBC (*n* = 224)	[96]
NOTCH	IHC	Notch 1 and Notch 3 expression correlated with poor prognosis (*n* = 126)	[97]
CD24	IHC	Positive expression was related to decreased OS (*n* = 207)	[98]
YAP1	IHC	High nuclear expression associated with poor survival in pT2 tumors (*n* = 132)	[99]
Msi-1 and ALDH1	IHC	Positive expression of Msi-1 or ALDH1 was an independent predictor of poor prognosis (*n* = 100)	[100]
MUC5AC	IHC	Reduced expression associated with decreased OS (*n* = 108)	[101]
ENT1	IHC	Low expression correlated with shorter median survival and lower OS (*n* = 214)	[102]
phospho-mTOR	IHC	High expression associated with poor prognosis in advanced GBC (*n* = 128)	[103]
lncRNA-LET	RT-qPCR	Low expression correlated with reduced metastasis free survival and OS (*n* = 128)	[104]
lncRNA-PAGBC	RT–qPCR	High expression associated with reduced OS (*n* = 77)	[105]
lncRNA-MEG3	RT-qPCR	Low levels correlated with a shorter OS (*n* = 50)	[106]
lncRNA-LINC01694	RT-qPCR	High expression correlated with a decreased OS (*n* = 40).	[107]
lncRNA-PVT1	ISH	High expression correlated with worse OS (*n* = 66).	[108]
serum VEGF-C	ELISA	High levels correlated with a shorter mean survival (*n* = 31 and 51 in each study).	[109,110]
serum IL-6	ECLIA	Low levels correlated with a better 5-year overall survival rate in a subgroup of patients with hepatic side tumors (*n* = 69).	[111]
preoperative NLR	Hemogram	High levels associated with lower median survival periods in GBC with hepatic involvement (*n* = 84).	[112]

Abbreviations: GBC, gallbladder cancer; OS, overall survival; IHC, immunohistochemistry; RT-qPCR, reverse transcription-quantitative polymerase chain reaction; ISH, in situ hybridization; ELISA, enzyme-linked immunosorbent assay; ECLIA, electrochemiluminescence immunoassay; NLR, neutrophil–lymphocyte ratio.

**Table 4:** Candidate predictive biomarkers for treatment selection in gallbladder cancer.

Biomarker	Measurement Technique	Scenario of Relevance and Relevance in Clinical Practice	Reference
Thymidine phosphorilase	IHC	Response to capecitabine/worse prognosis Relevance +	[128]
ERCC1/XRCC1	IHC	Response to gemcitabine/prognosis Relevance +	[129]
Cytidine deaminase	Sequencing; SNPs identification	Response to gemcitabine Relevance +	[139]
Circulating angiogenesis and inflammatory markers (i.e., VEGFR2)	ELISA	Prognosis on CisGem-treated patients. Relevance +	[140]
DNA Damage Repair deficiency	Sequencing/IHC	Response/prognosis in platinum-treated patients Relevance ++	[31,147,148,149,150,151]
TMB high/MMR deficiency/MSI high	Sequencing/IHC	Response to immunotherapy Relevance +++	[148,183,186,187,189]
HER2	Mutations (sequencing)	Precision medicine strategies (HER2 inhibitors) Relevance ++	[176,177]
BRAF mutations, RNF43 mutations, TRK-fusions	Mutations/fusions (sequencing)	Tumor agnostic precision medicine strategies Relevance +++	[161,162,163]

**Abbreviations:** IHC, immunohistochemistry; FISH, fluorescence in situ hybridization; SNP, single nucleotide polymorphism; CisGem, cisplatin and gemcitabine; TMB, tumor mutational burden; MMR, mismatch repair; MSI: microsatellite instability; DDR, DNA damage repair; +, little relevance; ++, significant relevance; +++, high relevance.
